# Disease-Causing Allele-Specific Silencing by RNA Interference

**DOI:** 10.3390/ph6040522

**Published:** 2013-04-11

**Authors:** Hirohiko Hohjoh

**Affiliations:** National Institute of Neuroscience, NCNP, 4-1-1 Ogawahigashi, Kodaira, Tokyo 187-8502, Japan; E-Mail: hohjohh@ncnp.go.jp; Tel.: +81-42-341-2711 (Ext. 5951); Fax: +81-42-346-1755.

**Keywords:** RNAi, allele-specific silencing, siRNA selection, dominantly inherited disease, nucleotide variation, allele discrimination, siRNA modification, forked siRNA

## Abstract

Small double-stranded RNAs (dsRNAs) of approximately 21-nucleotides in size, referred to as small interfering RNA (siRNA) duplexes, can induce sequence-specific posttranscriptional gene silencing, or RNA interference (RNAi). Since chemically synthesized siRNA duplexes were found to induce RNAi in mammalian cells, RNAi has become a powerful reverse genetic tool for suppressing the expression of a gene of interest in mammals, including human, and its application has been expanding to various fields. Recent studies further suggest that synthetic siRNA duplexes have the potential for specifically inhibiting the expression of an allele of interest without suppressing the expression of other alleles, *i.e.*, siRNA duplexes likely confer allele-specific silencing. Such gene silencing by RNAi is an advanced technique with very promising applications. In this review, I would like to discuss the potential utility of allele-specific silencing by RNAi as a therapeutic method for dominantly inherited diseases, and describe possible improvements in siRNA duplexes for enhancing their efficacy.

## Scientific Terms

*microRNA (miRNA)*: 21~23-nucleotide-long small non-coding RNA that functions as a mediator in transcriptional and post-transcriptional regulation of gene expression. MiRNA, like siRNA, is incorporated into RISC and works. Over 2000 miRNA genes have been found in the human genome
*“Seed” region*: nucleotides at positions 2-8 relative to the 5'-end of miRNA. The region is considered to be a key determinant of target specificity
*Short-hairpin RNA (shRNA)*: RNA sequence that forms a hairpin turn and can be processed by Dicer, an RNase III enzyme, to siRNA
*Allele*: one of a number of alternative forms of the same gene
*Single nucleotide polymorphism (SNP)*: single nucleotide variation, whose frequency in a population is more than 1%
*Induced pluripotent stem cell (iPSC)*: pluripotent stem cell artificially derived from a non-pluripotent cell

## 1. Introduction

RNA interference (RNAi) is the process of sequence-specific post-transcriptional gene silencing triggered by double-stranded RNAs (dsRNAs) homologous to silenced genes. This intriguing form of gene silencing has been found in various species including flies, worms, protozoa, vertebrates and higher plants [1,2,3,4,5]. Long dsRNAs (>30 bp), introduced or generated in cells, are processed by digestion with an RNase III enzyme, Dicer, into 21–25 nucleotide (nt) RNA duplexes [6,7,8,9]. The resultant RNA duplexes, referred to as small interfering RNA (siRNA) duplexes, are unwound and one of the siRNA strands of the duplexes can be incorporated into the RNA-induced silencing complex (RISC) and function as a sequence-specific RNAi mediator in the complex [6,9,10,11]; the siRNA strands incorporated into RISCs are referred to as guide siRNAs, and unincorporated strands are referred to as passenger siRNAs. Nucleotide sequences at positions 2–8 of guide siRNAs, corresponding to the “seed” region of microRNAs (miRNAs: endogenous, functional small non-coding RNAs which, like siRNAs, can be incorporated into RISCs) [12], presumably play an important role in the recognition of target RNAs. Argonaute2 (Ago2) is an essential component of RISC that directly associates with guide siRNA, and cleaves target RNAs at the phosphodiester bond that is across from nucleotide positions 10 and 11 of the guide siRNA [8,11,13,14,15,16,17,18,19,20,21].

In mammals, it was initially thought that RNAi might occur only in oocytes and preimplantation embryos [22,23,24]. Mammalian cells in general possess a rapid and nonspecific RNA degradation involving the sequence-nonspecific RNase, RNase L [25], and a rapid translation inhibition involving the interferon-inducible, dsRNA-activated protein kinase, PKR [26,27], both of which are activated by long dsRNAs (>30bp) and participate in an early defense system against virus infection in mammalian hosts. The rapid responses to long dsRNAs may mask RNAi triggered by the long dsRNAs in mammalian cells [28], except in the cases of undifferentiated cells [22,23,29,30] and differentiated cells that possibly lack PKR [31].

The discovery of chemically synthesized 21-nt siRNA duplexes capable of inducting mammalian RNAi without triggering the antiviral responses has become a major breakthrough [32] and has paved the way for a great advance in mammalian RNAi. Currently, RNAi can be induced in various kinds of mammalian cells, including human cells, by direct introduction of synthetic siRNA duplexes into cells or generation of siRNA duplexes using short-hairpin RNA (shRNA) expression vectors [33,34,35,36,37], some of which adopt the expression manner of miRNA genes.

In mammalian RNAi, different siRNAs, even though they target the same gene, show different levels of RNAi activities [38,39]; *i.e.*, RNAi activities depend upon the sequences of siRNAs used. Additionally, the relative thermodynamic stability of the termini of siRNA duplexes appears to influence unwinding of the duplexes, thereby possibly affecting RNAi activity [40,41].

The application of mammalian RNAi has expanded to several fields of science, of which the therapeutic use of RNAi in medical science and pharmacogenesis is particularly promising. In this review, I describe an advanced application of mammalian RNAi techniques for specifically inhibiting the expression of disease-causing alleles, and also suggest a possible modification of siRNAs to enhance such an RNAi for possible use as a new nucleic acid medicine.

## 2. Allele-Specific Silencing by RNAi As an Advanced Method for Therapeutic Use

Since RNAi was discovered [5], researchers have aimed at a nearly complete suppression of the expression of target genes by RNAi. Other than complete suppression, RNAi also has the potential for inhibiting the expression of an allele of interest without suppression of the expression of other alleles, *i.e.*, allele-specific silencing by RNAi or allele-specific RNAi (ASP-RNAi). This potential of RNAi appears to be dependent upon its highly sequence-specific knockdown manner, and can distinguish RNAi from conventional knockout methods.

**Figure 1:**
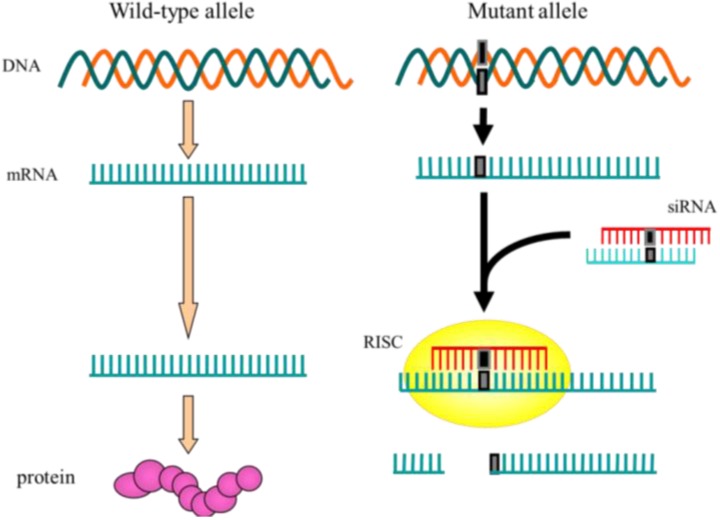
Outline of allele-specific silencing by RNAi. Wild-type and mutant alleles are indicated. Nucleotide variation (mutation) in the mutant allele and its transcript (mRNA) is indicated by solid box. siRNA duplex should be designed such that it can discriminate the mutant mRNA carrying the nucleotide variation that characterizes the mutant allele from wild-type alleles. After siRNA and RISC are assembled, the resultant RISC can exert allele-specific RNAi, *i.e.*, the RISC can preferentially recognize and cleave the mutant allele mRNAs, but not wild-type mRNAs, thereby decreasing the mutant product while the wild-type product remains stable.

ASP-RNAi is an advanced application of RNAi techniques, and likely to be therapeutically useful: it can specifically inhibit the expression of disease-causing alleles with minimal suppression of the expression from their corresponding wild-type alleles (Figure 1), *i.e.*, disease-causing allele-specific silencing by RNAi, or disease-causing allele-specific RNAi. Application of disease-causing allele-specific RNAi as a therapeutic method for dominantly inherited diseases such as familial amyotrophic lateral sclerosis, familial Alzheimer’s disease and Huntington’s disease appears to be particularly promising (see Table 1) [42]. In addition, disease-causing allele-specific RNAi can expect to provide an RNAi therapy possibly lacking adverse effects because of leaving the expression of wild-type alleles. As for a possible weak point, disease-causing allele-specific RNAi may remain inoperative as a therapeutic method for diseases caused by gene duplication mutation.

**Table 1:** Disease-causing allele-specific silencing by RNAi.

Disease	Target gene	Target variation	Inducer of RNAi	Competent siRNA(s) carrying mutation site(s) at the central position	Modification	References
Familial Alzheimer's disease	Amyloid precursor protein (APP)	K670N-M671L (Swedish mutant)	synthetic siRNA	yes	no	Miller VM. *et al.* (2004) [43] Ohnishi Y. *et al.* (2006) [44] Feng X. *et al.* (2006) [45]
	Amyloid precursor protein (APP)	K670N-M671L (Swedish mutant)	synthetic siRNA	no	nucleotide mismatches	Ohnishi Y. *et al.* (2008) [46]
	Amyloid precursor protein (APP)	V717F (London mutant)	synthetic siRNA	yes	no	Ohnishi Y. *et al.* (2006) [44]
	Amyloid precursor protein (APP)	V717I (London mutant)	synthetic shRNA	yes	no	Feng X. *et al.* (2006) [45]
	Preseniline 1 (PSEN1)	L392V	synthetic siRNA	yes	2-Thiouridine chemical modification	Sierant M. *et al.* (2011) [47]
Amyotrophic lateral sclerosis (ALS)	Superoxide dismutase (SOD1)	G93A	shRNA expression vector	yes	no	Xia X. *et al.* (2006) [48]
	Superoxide dismutase (SOD1)	G85R	synthetic siRNA	yes/no *1	nucleotide mismatch	Schwarz DS. *et al.* (2006) [49]
Slow channel congenital myasthenic syndrome (SCCMS)	Acetylcholine receptor (AChR)	aS226F	synthetic siRNA/shRNA	yes	no	Abdelgany A. *et al.* (2003) [50]
Frontotemporal dementia with parkinsonism linked to chromosome 17 (FTDP-17)	Microtubule-associated protein TAU (MAPT)	V337M	synthetic siRNA	yes	nucleotide mismatch	Miller VM. et al. (2003,2004) [43,51]
Ehlers-Danlos syndrome (vEDS)	Procollagen type III (COL3A1)	G252V	synthetic siRNA	yes	no	Muller GA. et al. (2012) [52]
Sickle cell anemia	Hemoglobin-beta locus (HBB)	E6V	synthetic siRNA	yes	no	Dykxhoorn DM. et al. (2006) [53]
Familial amyloidotic polyneuropathy (FAP)	Transthyretin (TTR)	V30M	synthetic siRNA	yes	no	Kurosawa T. et al. (2005) [54]
Fibrodysplasia ossificans progressiva (FOP)	Activin A receptor type I (ACVR1)	R206H, G356D	synthetic siRNA	yes	nucleotide mismatch	Takahashi M. et al. (2012) [55]
	Activin A receptor type I (ACVR1)	R206H	synthetic siRNA	yes	no	kaplan J. et al. (2012) [56]
Tumors	Phosphoinositide-3-kinase, catalytic, alpha polypeptide (PIK3CA)	1633G -> A 3140A -> G	synthetic siRNA	yes	no	Huang H. et al. (2009) [57]
Spinocerebellar ataxia type 1 (SCA1)	Ataxin-1 (ATXN1)	flanking region of expanded CAG repeat	shRNA expression vector	N/A *2	no	Xia H. et al. (2004) [58]
Machado-Joseph disease/spinocerebellar ataxia type 3 (MJD/SCA3)	ATAXIN3/MJD1	SNPs linked to expanded CAG repeat	synthetic siRNA / shRNA expression vector	yes	no	Miller VM. et al. (2003) [51] Alves S. et al. (2008) [59] Nobrega C. et al. (2013) [60]
Spinocerebellar ataxia type 7 (SCA7)	Ataxin-7 (ATXN7)	SNP linked to expanded CAG repeat	shRNA expression vector	no	no	Scholefield J. et al. (2009) [61]
Parkinson's disease	Leucine-rich repeat kinase 2 (LRRK2)	R1441G, R1441C	shRNA expression vector	yes	no	de Ynigo-Mojado L. et al. (2011) [62]
	Leucine-rich repeat kinase 2 (LRRK2)	G20195S	shRNA expression vector	no	no	Sibley CR. et al. (2011) [63]
	alpha-synuclein	A30P	shRNA expression vector	no	nucleotide mismatch	Sibley CR. et al. (2011) [63]
Huntington disease	Huntingtin (HTT)	SNPs linked to expanded CAG repeat	synthetic siRNA	yes/no *1	nucleotide mismatch	Pfister EL. et al. (2009) [64] Takahashi M. et al. (2010) [65]

*1: Not in some cases. *2: N/A, not applicable.

To induce disease-causing allele-specific RNAi, it is vital to design siRNAs or shRNAs that confer a strong allele-specific silencing, or allele-discrimination. Either siRNA or shRNA must be designed such that it can carry nucleotide variations characterizing target disease-causing alleles and can discriminate the target alleles from their corresponding wild-type alleles. Consequently, RISCs carrying the designed siRNAs can recognize and cleave only target mutant (disease-causing allele) RNAs, and neither recognize nor cleave wild-type allele RNAs. Ideally, the wild-type allele expression should be unchanged, but its reduction of 5%–10% may be tolerated in biological systems. Therefore, it may be an acceptable trade-off if the dominant disease-causing allele is potently silenced.

Single nucleotide polymorphisms (SNPs) as well as disease-causing nucleotide variations are capable of becoming targets against allele-specific siRNAs and shRNAs. The SNPs that lie in responsible genes for triplet-repeat diseases such as spinocerebella ataxia and Huntington’s disease appear to be particularly useful [51,59,60,61,65], *i.e.*, the SNPs linked with aberrantly expanded trinucleotide repeats in the disease-causing alleles are considered to be the same as disease-causing nucleotide variations. When SNPs are used as targets, SNP typing and linkage analysis between the SNPs and aberrantly expanded trinucleotide repeats must be performed in advance.

Current computer programs that can predict optimal conventional siRNAs from target gene sequences are less useful in predicting optimal allele-specific siRNAs. The prediction of optimal allele-specific siRNAs from target allelic sequences is quite difficult, or considered impossible. Therefore, siRNAs and shRNAs, designed for allele-specific silencing, must been examined one by one to see if they are capable of conferring allele-specific silencing.

## 3. Assessment of Allele-Specific RNAi

Mammalian RNAi activity depends upon the sequences of siRNAs used [38,39]. Similarly, different siRNA duplexes targeting the same allele appear to induce different levels of allele-discrimination, or allele-specific silencing. How can siRNA and shRNA, designed for ASP-RNAi, discriminate target (mutant) allele RNAs from wild-type allele RNAs? and, even though they may confer favorable cleavage of target mutant allele RNAs over wild-type allele RNAs, they may still retain some ability to cleave the wild-type allele RNAs; so, to what degree can designed siRNAs and shRNAs affect wild-type allele expression? These are major issues in performing ASP-RNAi, and hence it is of importance to determine optimal siRNAs and shRNAs for ASP-RNAi. Assessment of the effects of designed siRNAs and shRNAs on allele-specific silencing in a qualitative and quantitative manner is absolutely necessary. However, such an assessment is difficult. Selection of siRNAs and shRNAs may be addressed by conventional methods that are an independent assay, by which the effects of designed siRNAs and shRNAs on target mutant allele RNAs and wild-type allele RNAs are independently examined. In this case, a careful evaluation of normalization of the data obtained should be performed. Further advancement of the technique of allele-specific silencing most probably requires the establishment of a simpler yet precise assessment system. A heterozygous assay system with mutant and wild-type reporter alleles may be useful and applicable for selection of optimal siRNAs and shRNAs [44,66]. Briefly, the *Photinus* and *Renilla luciferase* reporter genes carrying mutant and wild-type allelic sequences in their 3'-untranslated regions are constructed as mutant and wild-type reporter alleles. The effects of designed siRNAs (or shRNAs) against mutant reporter allele in allele-specific silencing, as well as off-target silencing against wild-type reporter allele, can be simultaneously examined under a heterozygous condition generated by cotransfecting the reporter alleles and siRNAs (or shRNAs) into cultured mammalian cells. In either assay system, development of ASP-RNAi using cell-based reporters is a crucial first step, and demonstration experiments with affected patients’ cells [55,56,65] and model animals carrying human disease-causing alleles [59,60,67,68] need to be carried out. In addition, assessment of ASP-RNAi with iPSCs derived from affected patients [69] may be particularly promising in the future.

## 4. siRNAs and shRNAs Conferring Allele-Specific Silencing

ASP-RNAi targeting disease-causing alleles has been studied. Table 1 shows a summary of disease-causing allele-specific silencing by RNAi [43,44,45,46,47,48,49,50,51,52,53,54,55,56,57,58,59,60,61,62,63,64,65]. From the studies, it is suggested that either siRNAs or shRNAs possessing nucleotide mismatches against wild-type alleles at their central position had the potential for conferring allele-discrimination, or allele-specific silencing. Since active RISCs cleave target RNAs at the position corresponding to the center (between nucleotide positions 10 and 11) of the guide siRNA strand, it is conceivable that nucleotide mismatches that lie at the central position of siRNA would influence discrimination of target mutant RNA from wild-type allele RNA, and facilitate correct cleavage activity. Accordingly, the position of nucleotide mismatch(es) in guide siRNA against wild-type allele RNA is likely an important parameter for designing siRNA and shRNA conferring a potent allele-specific silencing.

## 5. Enhancement of Allele-Specific Silencing by Improved siRNA Duplexes

Enhancement of discrimination of target disease-causing allele RNAs from wild-type allele RNAs in ASP-RNAi is essential for further improvement of allele-specific silencing, and such an improvement of ASP-RNAi is under study. One possible way of enhancement of allele-specific silencing is to employ a modification technique. Various kinds of modifications can be incorporated into siRNA duplexes, and modified siRNAs appear to influence RNAi activities to various degrees. Of the modifications of nucleotides, the introduction of nucleotide changes (mismatches) into siRNA duplexes appears to be a simple yet effective method for influencing RNAi activity and also allele discrimination [46,49,55,57,65]. However, the following issues remain unsolved and are still unpredictable: which nucleotide position(s) of siRNA duplex in the introduction of mismatches is effective for enhancing ASP-RNAi?, and which nucleotide of three mismatched nucleotides should be selected?

A possible nucleotide position for introducing mismatches into siRNA duplexes may be the 3'-end of sense (passenger) siRNA strand. Forked siRNA duplexes, whose sense (passenger) strands carry a few nucleotide mismatches at the 3'- or 5'-ends against the antisense (guide) strands, appear to influence the selection of loading of siRNA strands into RISCs, thereby likely influencing RNAi activity [40,70,71]; and, forked siRNA duplexes carrying mismatches at the 3'-ends of the sense (passenger) strands may favorably load the antisense (guide) strands to RISCs. Ohnishi *et al.* (2008) [46] indicated that some forked siRNA duplexes carrying mismatches at the 3'-ends of the sense strands could improve ASP-RNAi activity.

Other nucleotide positions for introducing mismatches may also have the potential for influencing RNAi activity and allele discrimination [46,49,55,57,65]. To select optimal allele-specific siRNAs from variously designed siRNAs including mismatched siRNAs, we must assess those siRNA duplexes one by one for now. As an easy and precise assessment, the heterozygous assay system described above may work effectively [44,66].

Another possible improvement would be to introduce chemical modifications into siRNA duplexes. Sierant *et al.* (2011) [47] indicated that 2-thiouridine chemical modification introduced at the 3'-end of the antisense (guide)-stranded siRNA could improve ASP-RNAi activity. 5-bromo-uridine (U[5Br]), 5-iodo-uridine (U[5I]) and 2,6-diaminopurine (DAP) appear to increase the association constant between A-U base pairs [72]. Chiu and Rana (2003) demonstrated that synthetic siRNA duplexes carrying either U[5Br], U[5I], or DAP modification in guide siRNAs were able to induce RNAi activity, and suggested the possibility that the chemical modifications in guide siRNAs could increase the targeting efficiency of one RNA sequence over another closely homologous, but not identical, RNA sequence [72]. Other chemical modifications of ribonucleotides in siRNA duplexes such as 2'-*O*-methyl (2'-OMe), 2'-deoxy-2'-fluoro-β-D-arabino-nucleic acids (2'-FANA) and locked nucleic acid (LNA) also increase their resistance to ribonucleases such as serum-derived nuclease, resulting in increased longevity of RNAi activity [72,73,74,75,76]. Therefore, chemical modification of synthetic siRNA duplexes may be applicable for improvement of ASP-RNAi, both in terms of enhancing discrimination of allelic RNAs and increasing the persistence of ASP-RNAi activity. Taken together, it is suggested that chemical and/or structural modifications of siRNA duplex could be applicable to enhancing allele-specific silencing by RNAi.

Although we can hardly predict optimal allele-specific siRNAs from mutant and wild-type allelic sequences for now, the accumulation of data on allele-specific siRNAs and shRNAs will someday lead us to achievement of prediction and design of such optimal allele-specific siRNAs from target sequences. Therefore, more extensive studies on ASP-RNAi need to be carried out to control disease-causing allele-specific silencing in the future.

## 4. Summary

Allele-specific silencing by RNAi, or ASP-RNAi, is an advanced technique of RNAi that is likely to be therapeutically useful for dominantly inherited diseases such as neurodegenerative diseases and for complex diseases involving multiple genetic factors; namely, disease-causing allele-specific RNAi. To achieve ASP-RNAi, the following must be addressed: (i) selection of siRNA and shRNA that confer a strong allele-specific knockdown potency, and (ii) qualitative and quantitative assessment of the effects of those siRNAs and shRNAs on allele-specific silencing. Structural and chemical modifications of synthetic siRNA duplexes may be applicable for enhancement of allele-specific silencing by RNAi, and a simple evaluation system for ASP-RNAi may help for determining which particular siRNA and shRNA confer a strong allele-specific silencing. Such devised materials and methods could help contribute to the practical use of ASP-RNAi. To further control ASP-RNAi, a development of drug delivery system (DDS) for siRNA and shRNA is also vital. Therefore, more extensive studies including DDS studies need to be carried out to achieve clinical treatments with ASP-RNAi or RNAi.
